# A Lift-Off-Tolerant Magnetic Flux Leakage Testing Method for Drill Pipes at Wellhead

**DOI:** 10.3390/s17010201

**Published:** 2017-01-21

**Authors:** Jianbo Wu, Hui Fang, Long Li, Jie Wang, Xiaoming Huang, Yihua Kang, Yanhua Sun, Chaoqing Tang

**Affiliations:** 1 School of Manufacturing Science and Engineering, Sichuan University, Chengdu 610065, China; wujianbo@scu.edu.cn (J.W.); wangjie@scu.edu.cn (J.W.); 2016223020024@stu.scu.edu.cn (X.H.); 2 School of Mechanical Science and Engineering, Huazhong University of Science and Technology, Wuhan 430074, China; gm@chindt.com (L.L.); yihuakang@hust.edu.cn (Y.K.); yhsun@hust.edu.cn (Y.S.); 3 School of Electrical and Electronic Engineering, Newcastle University, Newcastle upon Tyne NE1 7RU, UK; c.tang2@newcastle.ac.uk

**Keywords:** MFL (magnetic flux leakage), drill pipe, lift-off distance, magnetic field focusing

## Abstract

To meet the great needs for MFL (magnetic flux leakage) inspection of drill pipes at wellheads, a lift-off-tolerant MFL testing method is proposed and investigated in this paper. Firstly, a Helmholtz coil magnetization method and the whole MFL testing scheme are proposed. Then, based on the magnetic field focusing effect of ferrite cores, a lift-off-tolerant MFL sensor is developed and tested. It shows high sensitivity at a lift-off distance of 5.0 mm. Further, the follow-up high repeatability MFL probing system is designed and manufactured, which was embedded with the developed sensors. It can track the swing movement of drill pipes and allow the pipe ends to pass smoothly. Finally, the developed system is employed in a drilling field for drill pipe inspection. Test results show that the proposed method can fulfill the requirements for drill pipe inspection at wellheads, which is of great importance in drill pipe safety.

## 1. Introduction

As critical components of drill strings, drill pipes are used to transmit torque and transport drilling fluids. During drilling operations, drill pipes withstand complex alternating stress loads, such as the pull, push, twist, and bend. In the meantime, drill pipes are also corroded by drilling fluids, which contain corrosive media such as dissolved O_2_, CO_2_, and H_2_S. As a result, cracks and corrosions easily develop in drill pipes, weakening the strength of drill pipes and even causing fracture failure [1]. Therefore, to avoid accidents and economic losses, according to the API (American Petroleum Institute) standard, drill pipes should be inspected by NDT (nondestructive testing) technologies before use [2]. MFL (magnetic flux leakage) technology is one of the most commonly used and powerful NDT methods and has been applied for the highly efficient inspection of defects in various kinds of ferromagnetic materials [3,4,5,6,7,8,9,10,11], particularly in elongated ferromagnetic objects, such as steel pipes [12,13,14,15,16,17] and wire ropes [18,19,20,21]. There are three types of MFL methods, namely, DC MFL, AC MFL, and pulsed MFL [22,23]. The DC MFL uses permanent magnets or direct currents to generate a static magnetizing field. Large direct currents can generate a strong magnetizing field; thus, the DC MFL based on direct currents is suitable for hard-magnetization objects, such as drill pipes. By applying an alternative current, the AC MFL is generally sensitive to surface defects due to the skin effect depending on the excitation frequency chosen, which is suitable for the detection of surface defects, such as surface cracks in steel bars. With pulsed MFL, the probe is driven with a pulsed current and the rich frequency components can provide information from different depths due to the skin effects. With proper signal processes and data analysis, additional information such as the location and size of defects can be obtained, which is suitable for precise inspection with a slow testing speed.

At present, after being removed from the well, drill pipes are usually transported to a far-away pipe station, then cleaned by high-pressure water, and thereafter inspected by automatic MFL testing equipment. In this way, the inspection requires a special testing site with a large area, and complicated hydraulic and mechanical systems, resulting in a considerable degree of inconvenience, i.e., high costs and significant time consumption. However, if the MFL inspection can be performed during the pulling movement of drill pipes out of the well, the operation will be greatly simplified, saving time and money, which is the main concern of this paper. To realize MFL inspection for drill pipes at wellheads, the main issue is how to inspect the soiled drill pipe surface with adhering mud, rock, and oil. In traditional MFL testing, in order to obtain higher sensitivity, magnetic sensors are positioned as closely as possible to the objects being tested, resulting in a near-zero lift-off value and a contact detection method [24]. The drawback is a short service life of contact probes caused by its severe wear, the need of repeated calibration due to the changes in lift-off distance generated by severe wear, and even probe damages caused by the high-hardness attachment on the pipe surface. As a result, a lift-off-tolerant MFL testing method is required, whereby the probes are positioned at a great lift-off distance but still have acceptable sensitivity.

There are several challenges in realizing lift-off-tolerant MFL testing for drill pipes at wellheads, which are as follows: (1) Normally, the drill pipes are hard to magnetize because they are made of high alloy materials and having high hardness after heat treatments. Magnetizing methods based on permanent magnets, alternative currents, and pulsed currents cannot meet the magnetizing requirements of the drill pipes at wellheads; hence, a strong magnetizing method is required; (2) The MFL sensor should have a sufficiently high sensitivity at a distance. Although there is a proposal available to improve the sensor SNR by the magnetic concentrating effect [17], the achieved lift-off distance of 3.0 mm is not enough for practical use. Meanwhile, the hall elements are easily saturated beyond working well under strong magnetic fields. Another high sensitive MFL method based on near-zero background magnetic fields is also not appropriate for drill pipes inspection at wellheads due to the structural restrictions of magnetic shielding devices [25]; (3) The complicated structure and serious swing movement of drill pipes should also be taken into consideration. In the automatic inspection, the probing system should pass though the large pipe ends smoothly without causing any mechanical collision. In addition, lift-off distance changes due to the swing movement of drill pipes should be avoided.

In this paper, to realize lift-off-tolerant MFL testing for drill pipes at wellheads, a novel MFL testing method that includes the Helmholtz coil magnetization method, the lift-off-tolerant MFL sensor, and the whole MFL inspection apparatus is proposed and developed here for the first time. Compared with the magnetization method based on permanent magnets, alternative currents, and pulsed currents [16,17,22,23], the Helmholtz coil method can generate a strong and uniform magnetizing field to meet the requirements of hard-magnetization drill pipes. The proposed MFL sensor is an active method that can guide more magnetic flux to leak out. It has high sensitivity at a distance and can be used for the contactless MFL testing. The developed MFL method is of great significance in drill pipe safety and can also be employed for other ferromagnetic material inspections at wellheads, such as those of casing pipes and tubing pipes.

## 2. The Scheme of the MFL Method at a Wellhead

It is well known that the MFL generated by the defects can be maximized only under the condition that the ferromagnetic object is fully magnetized to the saturation status. Drill pipes are made of high alloy materials and have a high hardness after heat treatment; thus, the drill pipes are hard to magnetize. Here, a Helmholtz coil magnetization method is proposed and designed. In this paper, the inspection for the most widely used 5 in. drill pipes (material grade: X80; thickness: 9.19 mm; body diameter: 127.0 mm; pipe end diameter: 184.2 mm) is analyzed and conducted. In the pulling operation, drill pipes usually randomly swing in the radial direction; hence, the magnetizing coil should have a large enough inner diameter for the large pipe ends to pass smoothly. The parameters of the Helmholtz coil magnetizer are as follows: the internal diameter of the coil is 284.0 mm, the external diameter of the coil is 375.2 mm, the thickness of the coil is 100 mm, the distance between the two coils is 100 mm, and each coil has 6000 ampere-turn, respectively.

**Figure 1:**
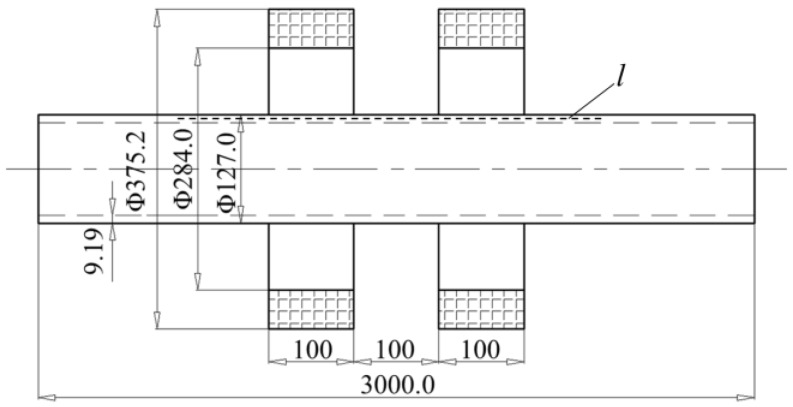
The simulation model for the 5 in. drill pipe magnetized by the Helmholtz coil (unit: mm).

**Figure 2:**
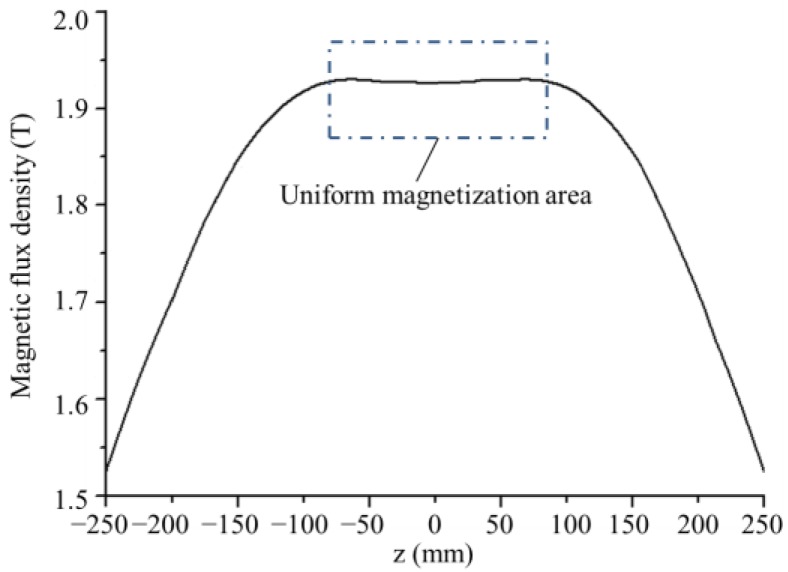
The axial magnetic flux density distribution in the drill pipe wall.

According to the magnetizer parameters and the X80 grade B-H curve, the magnetization of the drill pipe along line *l* is simulated by a numerical finite element method, as shown in Figure 1. Since the pipe and Helmholtz coil are axis-symmetric, finite element modeling and simulation procedures are implemented in 2D by the commercial electromagnetic simulation software ANSOFT. In mesh operation, the maximum length of elements is restricted to 1.0 mm. The axial magnetic flux density distribution from −250 mm to 250 mm is calculated and displayed in Figure 2. It can be seen that the drill pipe has been magnetized to saturation by the Helmholtz coils. In addition, a uniform magnetization area is formed in the pipe wall between the two coils, where the probing system can be placed.

**Figure 3:**
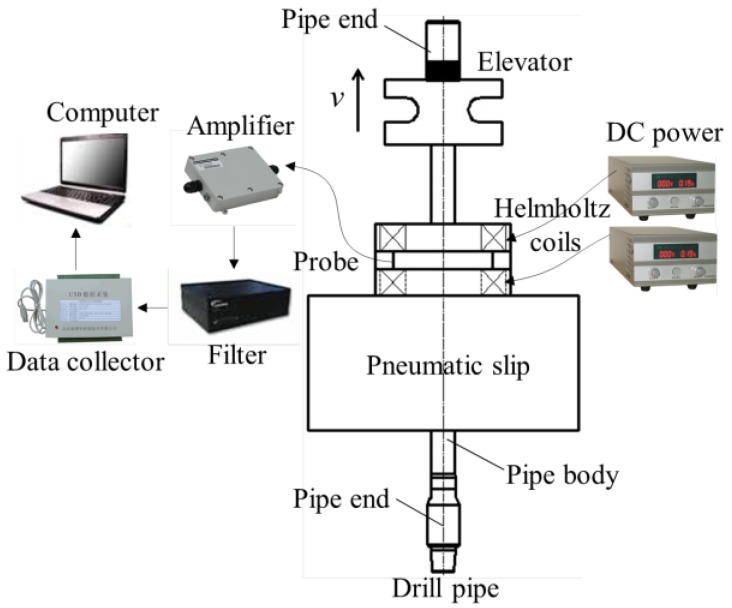
The diagram of the MFL (magnetic flux leakage) method for drill pipes at the wellhead.

Based on practical operation requirements at wellhead, the MFL method for drill pipes is proposed, as schematically illustrated in Figure 3. A pneumatic slip is used to hold the drill pipe when two connected drill pipes are separated from each other, and the pneumatic slip also provides a support for the magnetizer and probing system. The Helmholtz coil is placed on the pneumatic slip, which is driven by DC power. The MFL probing system is placed in the gap between the two magnetizing coils. When the drill pipe is being pulled out of the hole by the elevator, the probes scan the drill pipe automatically. If there are any defects in the pipe, MFL will be generated in the proximity of the pipe and detected by the probes. The analog signals from the probes are processed by the amplifier and filter, and the signals are then collected by a data collector and transformed into digital signals. Finally, the data are stored and analyzed by a computer.

## 3. The Lift-Off-Tolerant MFL Sensor Based on the Magnetic Field Focusing Effect

Due to the bad surface condition of drill pipes caused by the attachments such as mud, rock, and oil, the near-zero lift-off detection will evidently cause severe wear and even damage to the probe; as a result, a lift-off-tolerant MFL sensor at a great lift-off distance is required. Different from the method of building a near-zero background magnetic region with a magnetic shielding device [25], here, a lift-off-tolerant MFL sensor based on a magnetic field focusing effect is proposed.

**Figure 4:**
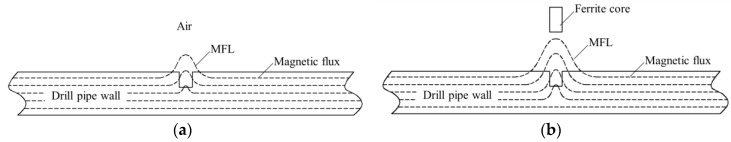
The principle of the lift-off-tolerant MFL sensing method based on the magnetic field focusing effect. (**a**) The MFL distribution without ferrite cores; (**b**) the MFL distribution with a ferrite core.

In the traditional MFL method, as schematically illustrated in Figure 4a, the magnetic flux leak into the air when the drill pipe is magnetized. Due to the lift-off effect, the leakage field rapidly decreases with increasing radial distance, leading to low sensitivity. However, as schematically depicted in Figure 4b, if a ferrite core is placed above the defect, more magnetic flux will be guided to leak into a larger space, forming a higher sensitivity at a distance. Basically, the magnetic field focusing effect is due to the high permeability of the ferrite core, and the “negative pressure” region caused by the ferrite core will guide more magnetic flux to leak out. In contrast with the traditional passive MFL sensing method, the proposed active method can allow the sensors to be placed at a greater lift-off distance.

**Figure 5:**
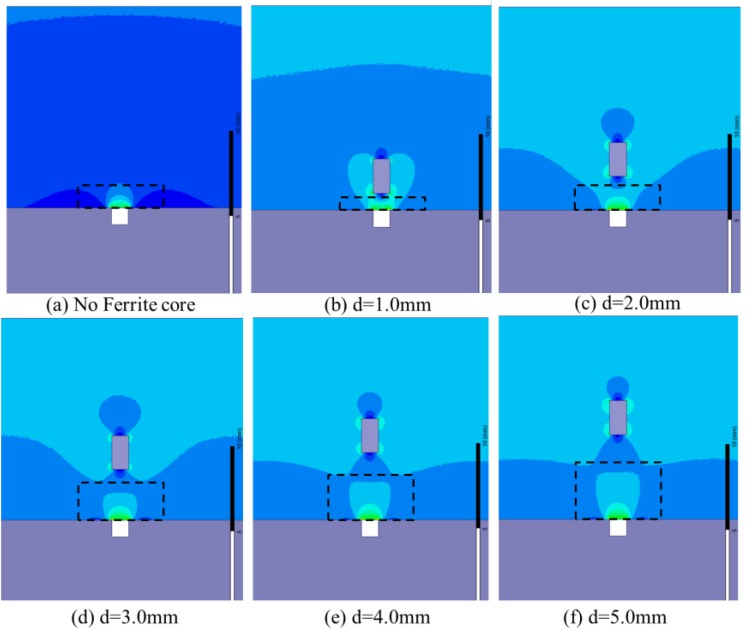
The distributions of the MFL affected by the ferrite core at different lift-off distances.

To investigate the magnetic field focusing process, finite element simulations of the MFL distribution affected by the ferrite core are performed. The same simulation parameters are used as displayed in Figure 1. A defect (1.0 mm in width and 1.0 mm in depth) is made in the drill pipe. The MFL distributions with a ferrite core (1.0 mm in width and 2.0 mm in height) positioned at different lift-off distances (1.0 mm, 2.0 mm, 3.0 mm, 4.0 mm, and 5.0 mm, respectively) are simulated, as shown in Figure 5. It can be seen that, owing to the magnetic field focusing effect of the high-permeability ferrite core, the magnetic flux are guided to leak into a larger space with the lift-off distance of the ferrite core increasing.

**Figure 6:**
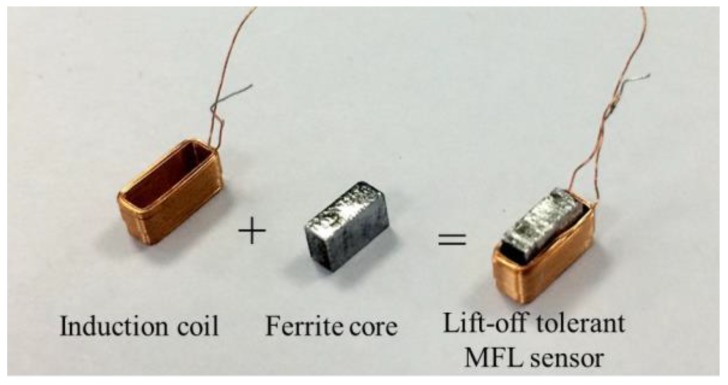
The diagram of the lift-off-tolerant MFL sensor.

Based the magnetic field focusing effect, a single lift-off-tolerant MFL sensor is developed, which consists of an induction coil and a ferrite core, as pictured in Figure 6. Specifically, the ferrite core is wound round by the insert coil made of enameled wire. Compared with hall elements, the induction coil can avoid the saturation problem under a strong magnetizing field.

**Figure 7:**
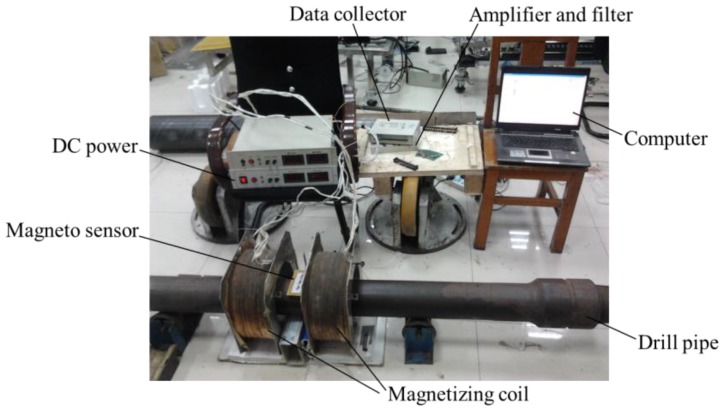
The experiment setup of the lift-off-tolerant MFL probe for drill pipes.

To compare the sensitivity of the lift-off-tolerant MFL sensor with the induction coil, the contrast experiment is conducted using a 5 in. drill pipe, as pictured in Figure 7. The experiment parameters are the same as the ones as displayed in Figure 1. A hole with a diameter of 1.6 mm is made in the drill pipe and scanned by the sensor placed at different lift-off distances of 1.0 mm, 2.0 mm, 3.0 mm, 4.0 mm and 5.0 mm, respectively. When the drill pipe is driven to move in the Helmholtz coil, the MFL is collected by the induction coil and the novel lift-off-tolerant MFL sensor, respectively.

**Figure 8:**
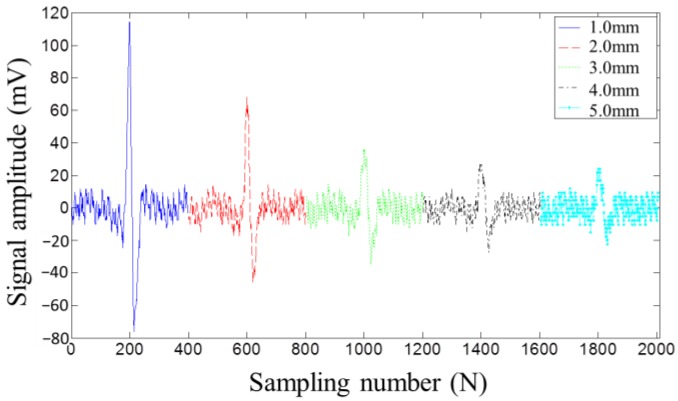
The MFL signals picked up by the induction coil at different lift-off distances.

**Figure 9:**
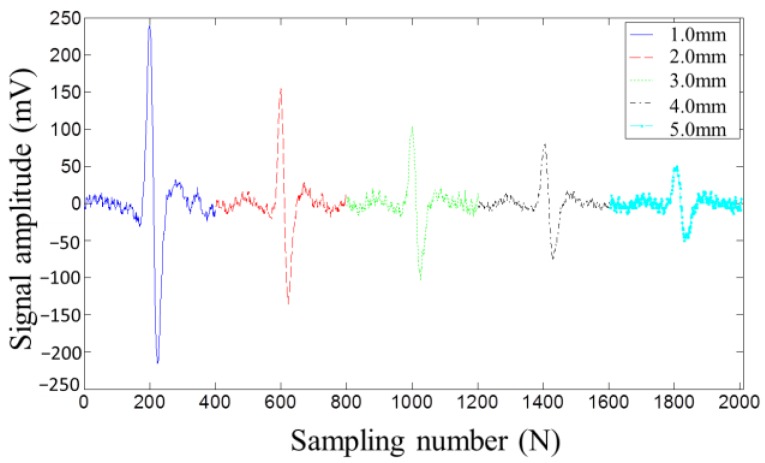
The MFL signals picked up by the lift-off-tolerant MFL sensor at different lift-off distances.

Figure 8 and Figure 9 display the MFL testing signals picked up by the induction coil and the lift-off-tolerant MFL sensor at the five lift-off distances, respectively. Both figures show that, with the lift-off distance increasing, the testing signal amplitude decreases. However, from Figure 8 and Figure 9, it can be seen that the signal amplitude picked up by the lift-off-tolerant MFL sensor is nearly twice the amplitude acquired by the induction coil at the same lift-off distance. In addition, the lift-off-tolerant MFL sensor shows a better SNR than the induction coil. From Figure 9, it can be seen that the novel sensor still shows high sensitivity at a lift-off distance of 5.0 mm, which can meet the requirements of the drill pipes inspection at wellheads. Thus, the lift-off distance of 5.0 mm is chosen as the optimal parameter for the lift-off-tolerant MFL sensor.

## 4. The Lift-Off-Tolerant MFL Probing System

**Figure 10:**
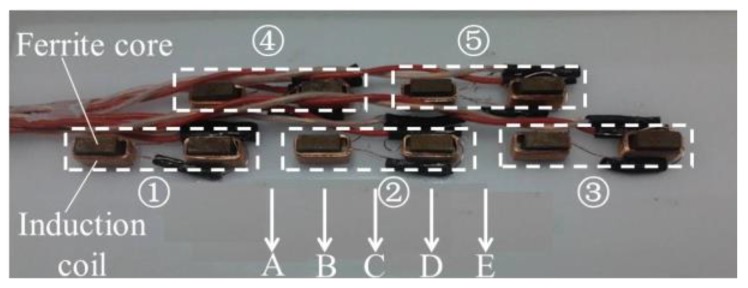
The sensor array in the lift-off-tolerant MFL probe in two layers.

Since a single MFL sensor has a limited sensing scope, in order to fulfill the 100% scanning coverage for drill pipes, the MFL probe embedded with the sensor array is required. Accurate evaluations are based on satisfactory repeatability, i.e., the same defect should generate the same MFL signal amplitude regardless of the path on which the defect passed through the probe. Here, the lift-off-tolerant MFL sensor array in two layers is designed, as shown in Figure 10. The signals picked up by two nearby sensors are added up as one channel, and there are ten sensors to form five channels (i.e., Channels ①, ②, ③, ④, ⑤). 

**Figure 11:**
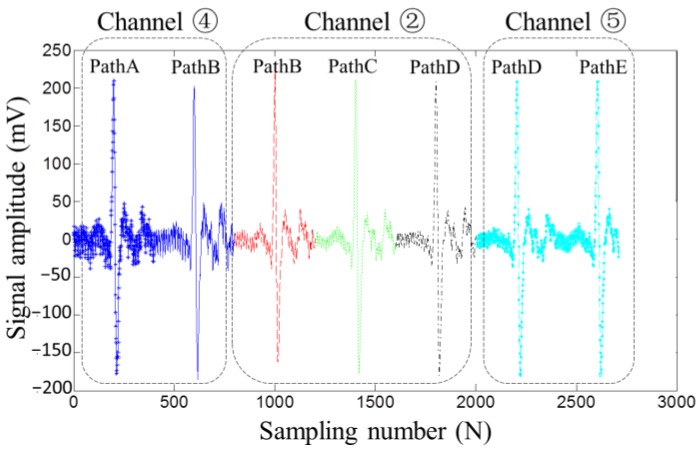
The MFL signals picked up by array lift-off-tolerant MFL sensors in different scanning paths.

To test the repeatability of the designed sensor array, using the same MFL experiment setup pictured in Figure 7, the hole is passed through the sensor array in different paths (Path A, Path B, Path C, Path D, Path E), as pictured in Figure 10. The testing signals picked up by Channels ②, ④, and ⑤ are displayed in Figure 11. It can be seen that, regardless of the path on which the defect passed through the sensor array, the constant largest amplitude is obtained by at least one channel, which proved that the proposed lift-off-tolerant MFL probes have good repeatability.

**Figure 12:**
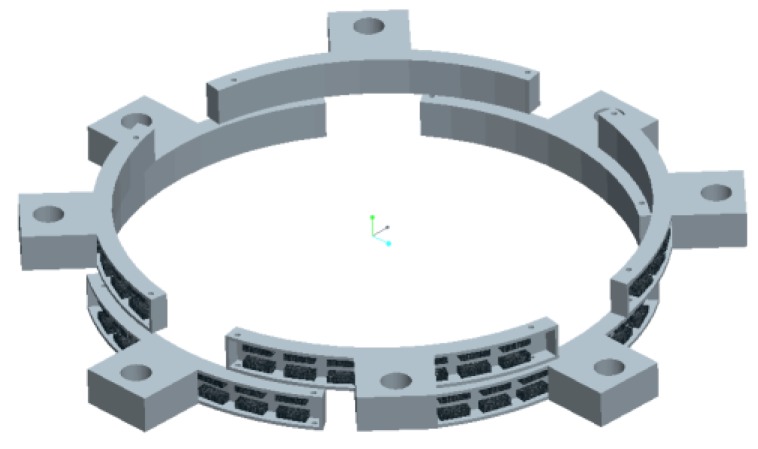
The MFL probing system integrated eight probes in two layers.

The MFL probing system integrated with eight probes is proposed, as schematically illustrated in Figure 12. The eight probes are arranged in two layers, and one layer is staggered ahead of the other one by 45°. If any defects pass through the gap between two nearby probes in one layer, it can be detected by the probes in the other layer. Each probe is embedded with 16 lift-off-tolerant MFL sensors, forming 8 channels. Thus, there are in total 64 channels for the MFL probing system.

**Figure 13:**
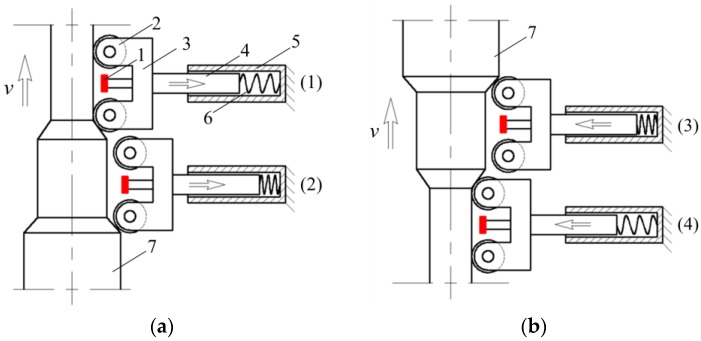
The follow-up scheme for the lift-off-tolerant MFL probing system. 1: probe; 2: wheel; 3: support; 4: slide; 5: guide rail; 6: pressure string; 7: pipe end. (**a**) the wheel rolling up the pipe end from Position (1) to Position (2); (**b**) the wheel rolling down the pipe end from Position (3) to Position (4).

Drill pipes inevitably swing during the pulling operation at the wellhead. To avoid the sensitivity change caused by the lift-off value change as displayed in Figure 9, the probes need to follow the pipe’s movement and keep a constant lift-off distance from the pipe surface. Besides, since pipe ends have a much larger diameter than the pipe body, the probing system should be sufficiently tolerant to allow the large pipe ends to pass smoothly without causing any damage to the probe itself. The follow-up scheme for the probing system was designed as schematically illustrated in Figure 13. The probe is fixed to a support, which is guided to move in the radial direction by the guide rail and slide. In the swing moment of the drill pipes, the wheel of the support is forced to contact the pipe surface firmly by the pressure string, forming a constant lift-off distance of the probe from the pipe surface. Besides, when the pipe ends arrive at the probing system, the wheel can firstly roll up and then roll down the pipe ends safely, as depicted in Figure 13a,b, respectively.

## 5. Application

**Figure 14:**
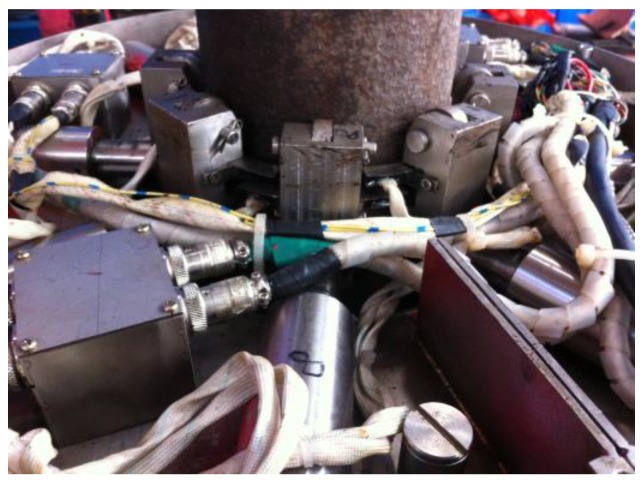
The lift-off-tolerant MFL probing system.

**Figure 15:**
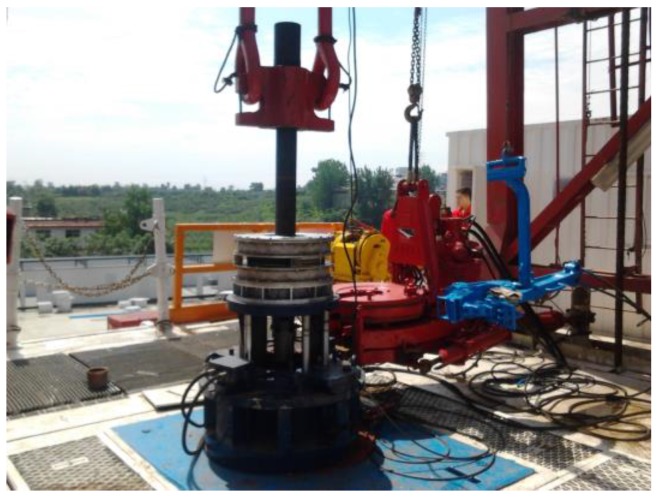
The whole MFL testing apparatus at the wellhead.

The lift-off-tolerant MFL system for 5 in. drill pipes at wellheads was employed in an actual drilling field. The probing system and the whole inspection apparatus are pictured in Figure 14 and Figure 15, respectively.

**Figure 16:**
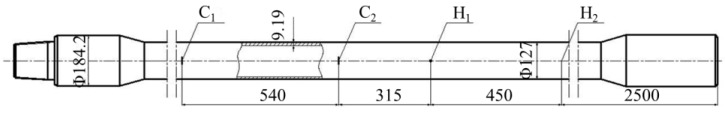
The drill pipe sample with four defects (unit: mm).

**Figure 17:**
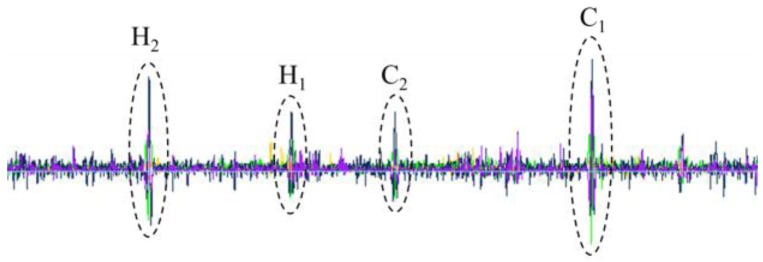
The typical MFL testing signals for the drill pipe at the wellhead.

In the test, a used 5 in. drill pipe is chosen as the testing sample. According to API Spec 5D, four standard defects are made in the drill pipe: C_1_ (circumferential crack with a width of 1.0 mm, a depth of 1.0 mm, and a length of 25.0 mm), C_2_ (circumferential crack with a width of 1.0 mm, a depth of 0.5 mm, and a length of 25.0 mm), H_1_ (hole with a diameter of 1.6 mm), and H_2_ (hole with a diameter of 3.2 mm), as depicted in Figure 16. When the drill pipe is being pulled out of the hole by the elevator at a speed of 20.0 m/min, it is magnetized by the Helmholtz coil and scanned by the MFL probes, and the typical testing signal is displayed in Figure 17. It can be seen that the MFL probes with a lift-off value of 5.0 mm have good sensitivity and satisfactorily meet API testing requirements.

**Figure 18:**
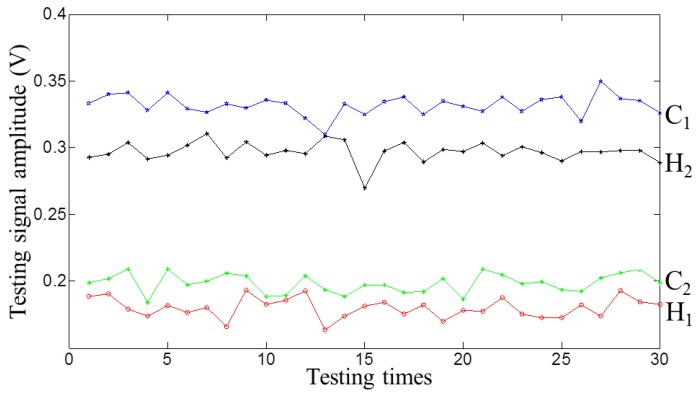
The testing signal amplitudes with the defects passed in different paths.

The repeatability of the MFL probing system was tested. The drill pipe was tested 30 times at a constant speed of 20.0 m/min. At each time, the entail probing system is rotated around the pipe axis 12° to ensure that the defect passed in different paths. The testing signal amplitudes for the four defects were recorded and are displayed in Figure 18. It can be seen that the MFL probing system has good repeatability.

## 6. Conclusions

To meet the great needs for MFL inspection of drill pipes at wellheads, a novel lift-off-tolerant MFL testing method has been proposed in this paper, which has high sensitivity and good repeatability. This novel method can also be used for other ferromagnetic material inspections at wellheads, such as those of casing pipes and tubing pipes. The following can be concluded.

(1) By applying a large direct current, the Helmholtz coil magnetizing method can generate a strong magnetic field to fully magnetize the drill pipe to the saturation status and form a uniform magnetization area in the pipe wall.
(2) Due to the high permeability of ferrite cores placed above the defect, more magnetic flux is guided to leak into a larger space, forming a higher sensitivity at a distance. In contrast with the traditional passive MFL sensing method, the proposed active method can allow the sensors to be placed at a greater lift-off distance.
(3) To fulfill the 100% scanning coverage for drill pipes, the sensor array in two layers and the probing system in two layers are proposed. Further, the follow-up device is designed to track the pipe’s movement and form a constant lift-off distance of the probes from the pipe surface. Tests results show that the probing system has a high sensitivity and good repeatability and allows the large pipe ends to pass smoothly.

## Supplementary Materials

The following are available online at http://www.mdpi.com/1424-8220/17/1/201/s1. Video S1: Video of the MFL inspection for drill pipes at wellhead.
